# Evolution of primate vocal repertoires: vocal systems as embodied capital for mediating within-group conflict

**DOI:** 10.1007/s10329-026-01271-2

**Published:** 2026-06-18

**Authors:** Eric Schniter, Mateo Peñaherrera-Aguirre

**Affiliations:** 1 Economic Science Institute, Chapman University, One University Drive, Orange, CA 92866 USA; 2 Center for the Study of Human Nature and Department of Anthropology, California State University, Fullerton, CA 92834 USA; 3 School of Animal and Comparative Biomedical Sciences, University of Arizona, Tucson, AZ 85721 USA

**Keywords:** Primates, Social complexity, Life history, Embodied capital, Macroevolution, Vocal communication, Vocal repertoire

## Abstract

Life history traits constrain investments in costly neural and somatic embodied capital, potentially shaping the evolution of primate vocal communication systems. This study integrates life history theory with the social complexity hypothesis to test a sequential pathway from longevity to social dynamics to vocal repertoire size, using updated vocal repertoire data for McComb & Semple’s (2005) set of 42 non-human primate species, alongside measures of group size and within-group conflict. We compiled data on vocal repertoire size, within-group conflict, group size, endocranial volume, and maximum longevity, analysing these via Phylogenetic Sequential Canonical Cascade Model and ancestral character reconstructions. Within-group conflict directly predicts vocal repertoire size, with earlier cascade steps—maximum longevity → endocranial volume → group size → within-group conflict—providing mechanistic support for this relationship. These findings suggest that embodied capital investments in neural and somatic traits enable conflict management across primate societies, with within-group conflict—itself scaled positively with group size—emerging as the proximal driver of vocal repertoire size, clarifying the coevolution of vocal systems and sociality across primates. This framework elucidates macroevolutionary patterns in nonhuman primates with relevance to hominin vocal complexity and language emergence.

**Supplementary Information:**

The online version contains supplementary material available at https://doi.org/10.1007/s10329-026-01271-2.

## Introduction

Phylogenetic and comparative studies of primate communication provide insights into the macroevolutionary processes that shaped vocal systems across the order and variation in their complexity (Lancaster 1968; Owren et al. 2011; Fedurek and Slocombe 2011; Nettle 2012). Communication transmits credible information from sender(s) to receiver(s), reducing receiver uncertainty, but requires transmitters’ and receivers’ benefits to outweigh costs for it to evolve (Darwin 1871, 1872; Owren and Rendall 2001; Maynard-Smith and Harper 2004; Scott‐Phillips 2008; Fischer and Price 2017).

Vocal systems entail both physical and functional costs in their growth, entrainment, and maintenance. Physical costs arise with the structures comprising these systems—organized neural tissue controlling vocal muscles and tracts, audition systems receiving sounds, and brain regions processing auditory signals. Functional costs involve using these structures to produce distinct, recognizable calls. Among primates, more capable systems are costlier to grow, develop, and maintain, often requiring extended maturation (Ekström 2024). We frame vocal systems as *embodied capital*: somatic investments in growth, development, and maintenance of traits like brain tissue or muscle control, which yield fitness returns over longer lifespans (Kaplan and Robson 2002; Kaplan et al. 2003). Standard life history theory explains lifespan and fertility variation (Cole 1954; Gadgil and Bossert 1970; Partridge and Harvey 1988), but embodied capital theory integrates this with investments in adaptations like neocortex for intelligence, visual displays, scent systems, or digestion (Kaplan et al. 2007)^1^.

Previous comparative studies have linked primate vocal repertoire size to social complexity, establishing the "social complexity hypothesis for communicative complexity"—the general thesis that more complex animal societies require more complex vocal systems with larger vocal repertoires to help regulate interactions among group members (Peckre et al. 2019; Aureli et al. 2022)^2^. Yet these relationships remain poorly understood because social complexity itself is a nebulous concept that has been inconsistently measured by variously examining group size and cohesion (McComb and Semple 2005; Nettle 2012; Kappeler 2019), dispersal and grouping patterns linked to mating system (Gustison et al. 2012), types and frequency of competition and conflict (Janson and Goldsmith 1995), dominance style (Kavanagh et al. 2021), and time spent grooming (McComb and Semple 2005; Arlet et al. 2015). Rather than treating social complexity as a single composite measure, we operationalize it through a mechanistic framework linking two key dimensions: group size and level of within-group conflict. Group size generates increased frequency of social encounters and competition for resources and mates, which in turn drives within-group conflict^3^. This operationalization allows us to move beyond descriptive characterizations of social complexity to test mechanistic predictions about how life history traits (via brain size and longevity) scaffold the social and communicative demands imposed by larger, more conflicted groups. Our perspective, informed by life history theory, extends the social complexity hypothesis by generating predictions about the ancestral emergence and phylogenetic distribution of primate vocal repertoires.

### The influence of life history and social factors on primate vocal repertoires

Relatively larger brains with expanded cortical association areas enable individuals to manage the ecological and social information critical for locating nutrient-dense resources such as ripe fruits, executing extractive foraging, and navigating group living challenges (Lancaster 1968; Owren et al. 2011; Fedurek and Slocombe 2011; Nettle 2012). Because brains are metabolically expensive to develop, mature, and maintain, animals with higher-quality diets tend to evolve larger brains with greater endocranial volume (Isler and van Schaik 2006). We propose that over primate evolutionary history, the costs of large brains paired with complex vocal systems featuring large repertoires have been offset by adaptive shifts in foraging strategies and social organization—specifically, living in groups that exploit patchy, ephemeral, and difficult-to-acquire resources like ripe fruits or mobile vertebrate prey (Kaplan et al. 2007).

Primate evolution exhibits two major transitions: the first, a gradual shift toward longer lifespans and greater embodied capital investments among most anthropoids, and the second, an intensification of this trajectory among some catarrhines. By enhancing foraging success and reducing predation risk, these adaptive shifts decreased extrinsic mortality (van Schaik and Isler 2012), enabling slower life histories with delayed reproduction and extended lifespans—conditions that permit substantial bioenergetic investment in costly embodied capital yielding delayed returns, including large brains and sophisticated vocal systems. Consequently, among primates, we expect maximum longevity to positively and significantly predict endocranial volume, and by extension, vocal repertoire size.

### Predictions and analytic framework

Drawing on this framework, we test four predictions: (P1) Maximum longevity positively predicts endocranial volume, as longer lives support costly brains (van Schaik and Isler 2012). (P2) Endocranial volume positively predicts group size, enabling navigation of larger groups with richer diets and social demands (Janson and Goldsmith 1995; Snaith and Chapman 2007; DeCasien et al. 2017; Dunn and Smaers 2018). (P3) Group size positively predicts within-group conflict (Lehmann and Boesch 2004; van Schaik 2016). (P4) Longevity, brain size, group size, and conflict positively predict vocal repertoire size.

Vocal repertoire size varies widely across primates and other taxa. McComb and Semple (2005) report that non-human primates produce 2–38 vocalizations primarily signalling traits and states, while human languages typically draw from 30–40 phonemes to build vast lexicons including abstract references^4^. Encephalization enables this complexity across multiple lineages—primates, cetaceans, corvids, elephants—via expanded motor cortex, though primates uniquely pair it with descended larynges (Reader and Laland 2002; Roth and Dicke 2005; Fitch 2010; Fitch et al. 2025).

Using literature data on 42 non-human primate species, we present descriptive statistics by clade (see Tables 1 and 2), Phylogenetic Sequential Canonical Cascade analysis, and ancestral reconstructions to examine evolutionary trends toward larger vocal repertoires, including those associated with the catarrhine shift. This work represents a major update to McComb and Semple’s (2005) foundational survey, incorporating two decades of subsequent vocal repertoire research. We first review descriptive statistics among various primate clades, then conduct a Phylogenetic Sequential Canonical Cascade analysis that examines the contribution of different predictors in a sequence of steps. Subsequently, we carry out ancestral character reconstructions to better understand the coevolution of social conflict and primate vocal repertoires over the course of primate evolution. Following presentation of our main results, we conclude by discussing implications of these findings for understanding vocal evolution in the hominid lineage and its potential relevance to language origins.

**Table 1:** Summary and sources of various indicators, factors, and controls used in analyses. Each social and life history variable corresponds to a category of traits hypothesized to influence vocal repertoire size

Variables	Categories	Source
Vocal repertoire size	Criterion	(McComb & Semple 2005) updates: (Batist et al., 2023; Bosshard et al., 2024; Bouchet et al., 2010; Crockford, 2019; Erb et al., 2024; Fichtel & Kappeler, 2022; Gamba et al., 2015; Girard-Buttoz et al. 2025; León et al., 2014; Lopes, 2020; Muir et al., 2025; Nadhurou et al., 2016; Paulo-Morelo & Sánchez-Palomino, 2021; Salmi et al., 2013; Wegdell et al., 2025)
Group size	Social	(Jones et al. 2009)
Within-group conflict	Social	(Plavcan et al. 1995; Plavcan2001, see text for further details)
Endocranial volume	Life history	(DeCasien et al. 2017; Herculano-Houzel 2019; van Shaik 2012)
Maximum longevity	Life history	(Jones et al. 2009)
Body size	Life history	(Jones et al. 2009)
Research effort	Zoological record	(Reader et al. 2011)

Data available online at Schniter E, Peñaherrera-Aguirre M (2026) Data Associated with Manuscript Titled “Evolution of Primate Vocal Repertoires: Vocal Systems as Embodied Capital for Mediating Within-group Conflict”. [Data set]. Zenodo. https://doi.org/10.5281/zenodo.18291582.

**Table 2:** Descriptive Statistics of maximum longevity, endocranial volume, group size, within-group competition, and repertoire size aggregated at the suborder, infraorder, superfamily, and family level

Clade	Statistic	Maximum longevity (years)	Endocranial volume	Group size	Within-group conflict	Repertoire size
Hominid	M	55.500	404.013	37.800	0.084	13.400
	SD	5.745	71.641	31.499	0.737	3.362
	n	4	4	4	4	4
Cercopithecoid	M	31.750	89.004	27.252	0.630	17.267
	SD	6.401	29.445	20.012	0.646	6.352
	n	15	15	15	15	15
Platyrrhine	M	24.591	29.436	12.209	-0.127	15.818
	SD	7.091	31.671	11.123	0.725	6.431
	n	11	11	11	11	11
Strepsirrhine	M	23.358	17.484	4.942	-0.700	10.917
	SD	8.713	13.124	4.705	0.846	6.112
	n	12	12	12	12	12

Taxonomic groupings (suborder, infraorder, family) reflect the branching structure of the primate phylogeny and are used here as a reader-oriented organizational tool to situate species within their evolutionary context

## Methods

### Sample and measures

#### Vocal repertoire size

We collected vocal repertoire size data from McComb and Semple (2005), who operationalized a species’ vocal repertoire as the set of acoustically discrete calls reliably developing among adults. Their sample excluded infant and juvenile vocalizations, combinatorial vocalizations including gibbon song, and other sounds such as teeth chattering and grinding, hissing, and lip smacking. To update these data, we systematically searched the primatological literature for studies published after the sources cited by McComb and Semple (2005) providing revised repertoire counts for the 42 species in their dataset. We adhered strictly to McComb and Semple’s criteria when evaluating these updates and revised taxonomic names to reflect current classifications where applicable. Thirteen of 42 species received numeric updates relative to McComb & Semple (2005); the remaining 29 species retained their original values, confirmed by post-2005 literature or the absence of qualifying post-2005 studies. Among the 13 updated species, 8 decreased and 5 increased (mean McComb & Semple count: 18.1; mean updated count: 12.6). We note that the *P. pygmaeus* value of 10 that we use, from McComb & Semple (2005), was derived primarily from observations of Bornean orangutans by MacKinnon (1974) — a study that predates the current species-level split between P. pygmaeus and P. abelii — and that a systematic field-based adult repertoire study for Bornean orangutans conforming to standard comparative criteria remains a gap in the literature. Decreases predominate among species whose original counts were derived from captive populations or from observer-based classification methods now understood to over-split acoustically graded call variants into separate types. Post-2005 quantitative acoustic analyses, many employing machine learning classification of spectrograms, group calls by acoustic similarity rather than behavioral context, yielding smaller discrete type counts for these species. Increases occur for species whose older qualitative field descriptions undercounted call types subsequently documented by detailed acoustic analysis. The bidirectionality of updates reflects species-by-species evidence-following rather than a systematic directional correction. Both the original McComb and Semple (2005) repertoire counts and our updated dataset are provided in an online supplement (Schniter & Peñaherrera-Aguirre 2026). To assess robustness, we replicated all analyses using the original McComb & Semple (2005) vocal repertoire counts in place of our updated values; results are reported in the Appendix.

#### Social variables

Species’ group size data, defined as the total number of individuals (adults, subadults, and immatures) comprising the stable social community within which individuals regularly maintain social relationships, were collected from the *PanTHERIA* database (Jones et al. 2009). This is appropriate because group size here functions as a measure of the overall social environment within which adults interact and compete; the presence of immatures affects adult proximity patterns, resource competition, and conflict dynamics. The adult restriction applied to vocal repertoire counts (following McComb & Semple 2005) is a methodological criterion for the outcome variable ensuring species-typical call representation, and does not imply a corresponding restriction on the group size predictor. We use community-level group size rather than co-present party size because within-group conflict potential scales with the full set of social relationships an individual must manage – including those with members encountered intermittently in fission–fusion societies and not only those co-present at a given moment (Lehmann and Boesch 2004; van Schaik 2016; Dunbar and Shultz 2021). For most species in our dataset, PanTHERIA values approximate community-level census size; for fission–fusion species where PanTHERIA reflects mean party size, we supplemented with community-level estimates from sources that explicitly correct for this conflation (see below). We also gathered group size data for *Chlorocebus aethiops* from the online database *All the World’s Primates* (Rowe & Myers, https://alltheworldsprimates.org/).

For Pongo pygmaeus, the original group size value of 2 (Rowe 1996, as reported in McComb & Semple 2005) reflects mean party/encounter size for a largely semi-solitary species and is therefore not an appropriate measure of community size. We replaced this value with the local community size estimate of 14 from Dunbar et al. (2018), whose dataset was specifically constructed to use community-level rather than party-level group size estimates across primates. This is consistent with the approach recommended by Dunbar & Shultz (2021), who explicitly identify Pongo as a species for which local community size must be used in place of foraging party size to avoid systematic error in comparative analyses.

Data on species’ frequency of within-group conflict and intensity of within-group conflict, and the presence of coalitions and alliances were collected from Plavcan and colleagues (1995) and Plavcan (2001). Plavcan’s datasets are classified into binary variables (0: absence; 1: presence) including: (1) male intensity of conflict; (2) female intensity of conflict; (3) male frequency of conflict; (4) female frequency of conflict; (5) male coalitions and alliances; (6) and female coalitions and alliances. We generated three indices by aggregating the male and female indicators per variable, producing the following continuous variables: (1) male within-group of conflict; and (2) female within-group conflict. Additional data on within-group conflict indicators were gathered from Campbell et al., (2007), Díaz-Muñoz et al. (2011), Romano et al., (2019), Setchell et al., (2006), and Yamamoto et al., (2014). All conflict variables were later used as part of a unit-weighted factor estimation procedure described in more detail below.

#### Life history variables

Data on species’ adult endocranial volume (ECV) were collected from DeCasien et al (2017), Herculano-Houzel (2019), and van Shaik & Isler (2012) with preference given to DeCasien et al. (2017) and Herculano-Houzel (2019) where values were available across multiple sources. This hierarchy accounts for the fact that values from these sources are occasionally larger than those reported by van Schaik & Isler (2012) alone — for example, our values of approximately 505 cm^3^, 394 cm^3^, and 381 cm^3^ for *Gorilla gorilla*, *Pan troglodytes*, and *Pongo pygmaeus*, respectively, reflect the DeCasien et al. (2017) and Herculano-Houzel (2019) compilations rather than van Schaik & Isler’s (2012) figures of 433, 356, and 337 cm^3^. These differences reflect source-level variation in measurement method and specimen sampling rather than errors in data entry, and we have verified our values against the primary sources used. This variable was later log-transformed (ln) prior to analysis.

Maximum longevity and body mass (g) were sourced uniformly from the PanTHERIA online data repository and log-transformed (ln) prior to analysis. PanTHERIA was constructed specifically for cross-species macroecological and macroevolutionary analyses and is the field-standard source for this type of comparative study. Maximum longevity is the preferred metric in comparative primate life-history and phylogenetic analyses (Jones et al. 2009; De Magalhães and Costa 2009) because it reflects evolved intrinsic somatic-maintenance potential under low extrinsic mortality — the upper bound that co-evolves with costly investments in brain size and social complexity — rather than average lifespan, which is heavily confounded by ecologically variable juvenile and extrinsic mortality irrelevant to adult traits. We note that PanTHERIA values may be conservative for some species relative to more recent records (e.g., 60 years for *Pan troglodytes* vs. 66 years reported by Wood et al. 2017 and a captive maximum of 68 years); however, methodological consistency across all 42 species requires a single standardized source, as selectively substituting newer species-specific estimates for individual taxa would introduce heterogeneous data provenance. Because all PSCCM variables are z-standardized prior to analysis, modest differences in a single species’ longevity value have negligible quantitative effect on results. Future work using updated databases such as AnAge (de Magalhaes & Costa 2009) would be a worthwhile refinement.

We estimated the expected longevity value for *Piliocolobus badius* by examining the association between maximum longevity and age at first birth (also sourced from PanTHERIA). We compared the taxonomic classification of *Piliocolobus badius* across databases and detected no noticeable discrepancies. As species-level values may reflect research effort, we collected data on the number of zoological records published per species from Reader and colleagues (2011) and residualized species-level variables for research effort in all regression analyses. Due to the unavailability of a robust gut morphology dataset for our full 42-species sample, we conducted a supplementary analysis of gut morphology as a life history trait to explore dietary quality’s role in embodied capital trade-offs (see Appendix).

### Unit-weighted within-group conflict factor estimation

In contrast to traditional latent dimension procedures (e.g., principal axis factor analysis or principal component analysis) requiring larger sample sizes, unit-weighted factor estimation (UWF) provides a powerful analytical tool for computing and evaluating a latent structure. We computed the UWF score with the primates’ within-group conflict indicators as the arithmetic means of standardized indicators (*z*-values). The bivariate part-whole correlations between UWF scores and *z*-values are mathematically equivalent to factor loadings (Figueredo et al. 2017). All statistical analyses were conducted in R v 4.0.1 (R Core Team 2021). To support the factor structure estimated using the latter procedure, phylogenetic independent contrasts were computed (using the ape package; Paradis and Schliep 2019) and later analysed using Horn’s parallel analyses, to determine the number of recommended factors. Following this examination, a Principal Axis Factor analysis was computed following the number of recommended dimensions using the corresponding phylogenetic independent contrasts. These latent dimension analyses were conducted using the psych package (Revelle 2024).

### Phylogenetic sequential canonical cascade models

**Fig. 1:**
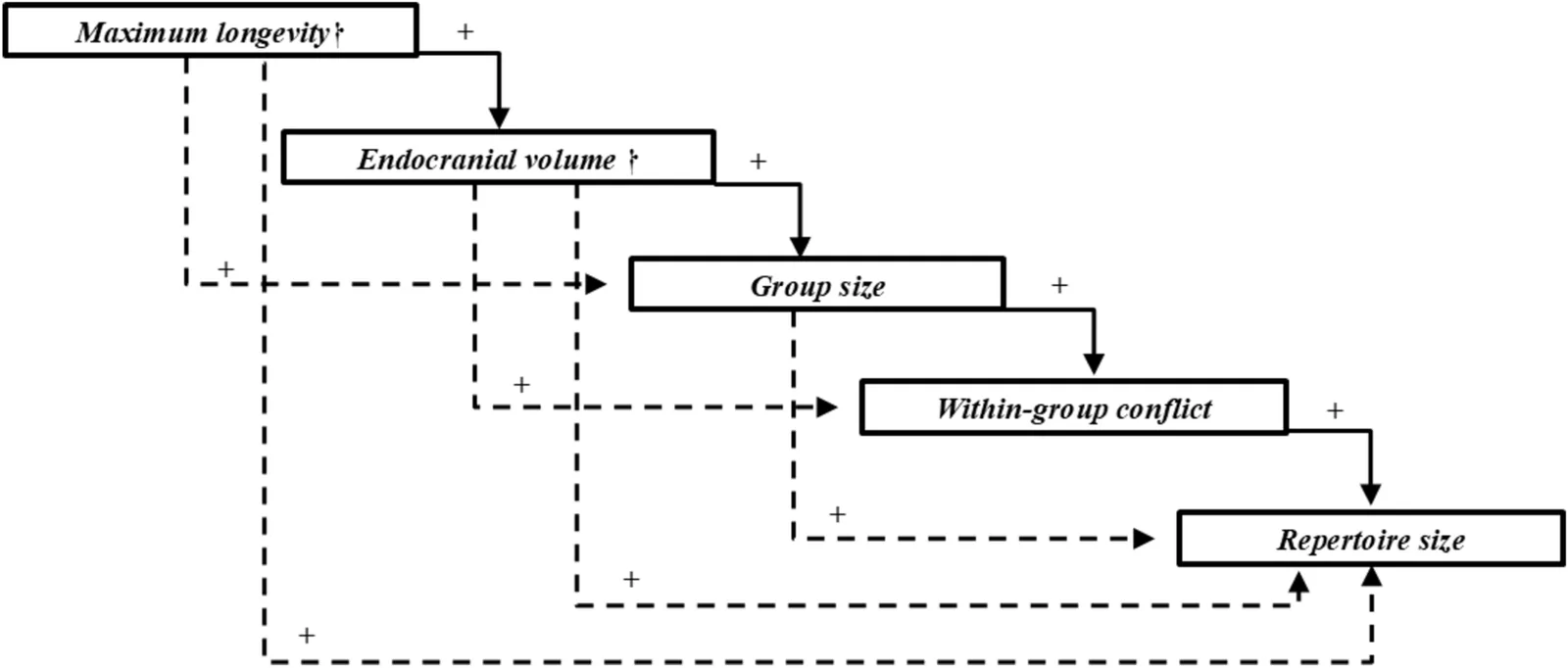
Simplified diagram of a predicted Phylogenetic Sequential Canonical Cascade model examining the direct and indirect influence of *maximum longevity*, *endocranial volume*, *group size*, and the *within-group conflict* on the *vocal repertoire size*. The dagger symbol () indicates the variable is also residualized for log (ln) body mass. All variables were residualized for research effort

For this study, we used a phylogenetic sequential canonical cascade model (PSCCM) to explore hierarchical relationships among predictors while minimizing Type I errors through sequential variance partitioning (Figueredo 2013; Figueredo et al. 2017). PSCCMs extend hierarchical general linear modelling by treating each dependent variable as a predictor in subsequent regressions. Unlike path analysis or structural equation modelling (SEM), which estimate parameters simultaneously, PSCCM’s sequential approach is ideal for exploratory analyses before confirmatory testing. Each step includes: (1) an omnibus test (*F*-value and *p*-value); (2) the model’s *R*^*2*^; and (3) Pagel’s *λ*, with likelihood ratio tests to assess phylogenetic signal (0 for non-conserved residuals, 1 for conserved residuals). We examined five variables, residualized for research effort: (1) *maximum longevity*, (2) *endocranial volume*, (3) *social group size*, (4) *within-group conflict*, and (5) *vocal repertoire size*. *Maximum longevity* and *endocranial volume* were also residualized for body mass, as described below. Figure 1 illustrates the proposed sequence.

Because the PSCCM tests each link in the causal chain sequentially rather than estimating direct and indirect effects simultaneously, it does not constitute formal mediation testing in the structural equation modelling sense. It is, however, specifically designed to generate evidence consistent with sequential mediation: at each step, distal predictors are tested for residual direct effects on the criterion once the proximal predictor is included, and the pattern of which variables lose predictive value at which steps provides exploratory support for a mediating causal structure (Figueredo 2013; Figueredo et al. 2017). We use mediation language throughout in this exploratory sense.

To develop body mass controls, a general linear model was computed between the species’ maximum longevity and log body mass. From this, standardized residuals were extracted. These residuals were next used as a criterion variable in a general linear model with research effort as a predictor, from which the residuals were extracted. A general linear model was also computed between the species’ endocranial volume and log body mass, from which standardized residuals were extracted. In turn, these residuals were used as criterion variables in a general linear model with research effort as a predictor, from which the residuals were extracted. A similar analysis was conducted using the original McComb and Semple database (2005).

### Ancestral character reconstructions

We obtained the phylogenetic tree for the primate species in our sample from the 10kTrees website (Arnold et al. 2010; https://10ktrees.nunn-lab.org/Primates/downloadTrees.php). This consensus tree, estimated from 10,000 phylogenies with branch lengths generated via Bayesian inference, follows a chronogram structure, with branch lengths proportional to time. We downloaded this tree, loaded it into R, and overlaid each trait onto the phylogeny for ancestral reconstruction. The reconstructed values reflect the time estimates derived from the consensus tree’s underlying phylogenies.

We use ancestral character reconstructions (ACR) via maximum likelihood estimations to visually inspect the macro-coevolutionary patterns between primate vocal repertoire sizes and within-group conflict, both at the level of the general latent dimension as well as at the level of the factor’s indicators. We performed the ACRs using the anc.ML function found in package phytools (Revell 2012).

### Ethical note

No ethical clearance was required for this study.

## Results

Our analyses confirm the predicted relationships among life history traits, social factors, and vocal repertoire size, with within-group conflict acting as the proximal predictor in a sequential cascade consistent with a mediating role between group size and vocal complexity. We report statistical values for maximum longevity, endocranial volume, social group size, within-group conflict, and vocal repertoire size across nonhuman primates, aggregated at select suborders (Strepsirrhini), parvorders (Platyrrhini), superfamilies (Cercopithecoidea), and family levels (Hominidae, Lemuridae, Lorisidae, Callitrichidae, Galagidae) in Tables 2 and 3. Relative to mean values of other primate taxa examined, hominids in our sample have longer lifespans (Hominidae M = 55.50, SD = 5.75) and larger endocranial volumes (Hominidae M = 404.01, SD = 71.64) than other taxa (see Table 2), while cercopithecoids show larger repertoires (Cercopithecoidea M = 17.27, SD = 6.35; Table 2). Their group sizes (Hominidae M = 35.5, *SD* = 39.669) were also larger than comparison taxa (Cercopithecoidea M = 27.252, *SD* = 20.012, Platyrrhini M = 12.209, *SD* = 11.123, Strepsirrhini M = 4.942, *SD* = 4.705), though certain cercopithecoid species, particularly within *Papio* and *Theropithecus* genera, can form groups that approach or exceed those of hominids (Dunbar and Shultz 2021). In our sample, non-human primates in the Hominidae family displayed within-group conflict factor scores near the primate mean, with values higher than those observed in species from Lemuridae, Lorisidae, Callitrichidae, and Galagidae families (Table 3).

**Table 3:** Descriptive Statistics of maximum longevity, endocranial volume, group size, within-group competition, and repertoire size aggregated at the family level

Family	Statistic	Maximum longevity (years)	Endocranial volume	Group size	Within-group conflict	Repertoire size
Atelidae	M	27.500	76.191	23.050	0.184	15.000
	SD	3.536	34.137	14.071	0.684	9.899
	n	2	2	2	2	2
Callitrichidae	M	20.367	9.352	6.492	− 0.299	17.000
	SD	4.260	3.120	1.351	0.617	6.663
	n	6	6	6	6	6
Cebidae	M	34.000	48.473	23.150	0.651	16.000
	SD	9.899	34.307	16.546	0.023	7.071
	n	2	2	2	2	2
Cercopithecidae	M	31.750	89.004	27.252	0.630	17.267
	SD	6.401	29.445	20.012	0.646	6.352
	n	15	15	15	15	15
Galagidae	M	14.500	4.824	4.000	− 1.142	9.250
	SD	2.082	1.365	2.273	0.250	5.965
	n	4	4	4	4	4
Hominidae	M	55.500	404.013	37.800	0.084	13.400
	SD	5.745	71.641	31.499	0.737	3.362
	n	4	4	4	4	4
Lemuridae	M	31.800	24.967	8.060	-0.006	15.600
	SD	3.033	3.885	0.666	0.939	4.037
	n	5	5	5	5	5
Lorisidae	M	19.500	10.119	1.000	− 1.267	3.500
	SD	9.192	4.237	0.000	0.000	2.121
	n	2	2	2	2	2

### Unit-weighted within-group conflict factor

Prior to estimating the PSCCM, it was pertinent to determine the statistical properties of the within-group conflict factor. The unit-weighted estimations support the presence of this general latent dimension. The unit-weighted factor loads positively and significantly onto the male within-group conflict and female within-group conflict indicators (factor *λ* = 0.892, *p* < 0.0001). Additionally, phylogenetic independent contrasts were computed on male within-group conflict and female within-group conflict indicators. Horn’s parallel analysis recommended the extraction of a single latent dimension (resampled data eigenvalue = 0.23, simulated eigenvalue = 0.27, actual data eigenvalue = 1.068). A Principal Axis Factor (PAF) analysis was computed on these phylogenetically-independent values. The PAF supported the presence of a within-group conflict factor that explained 53.4% of the variance and loaded positively and sizably onto the latter conflict indicators (factor *λ* = 0.731,* p* < 0.0001). Consequently, the latter results support the factor structure estimated using unit-weighted estimations.

### Phylogenetic sequential canonical cascade models

**Fig. 2:**
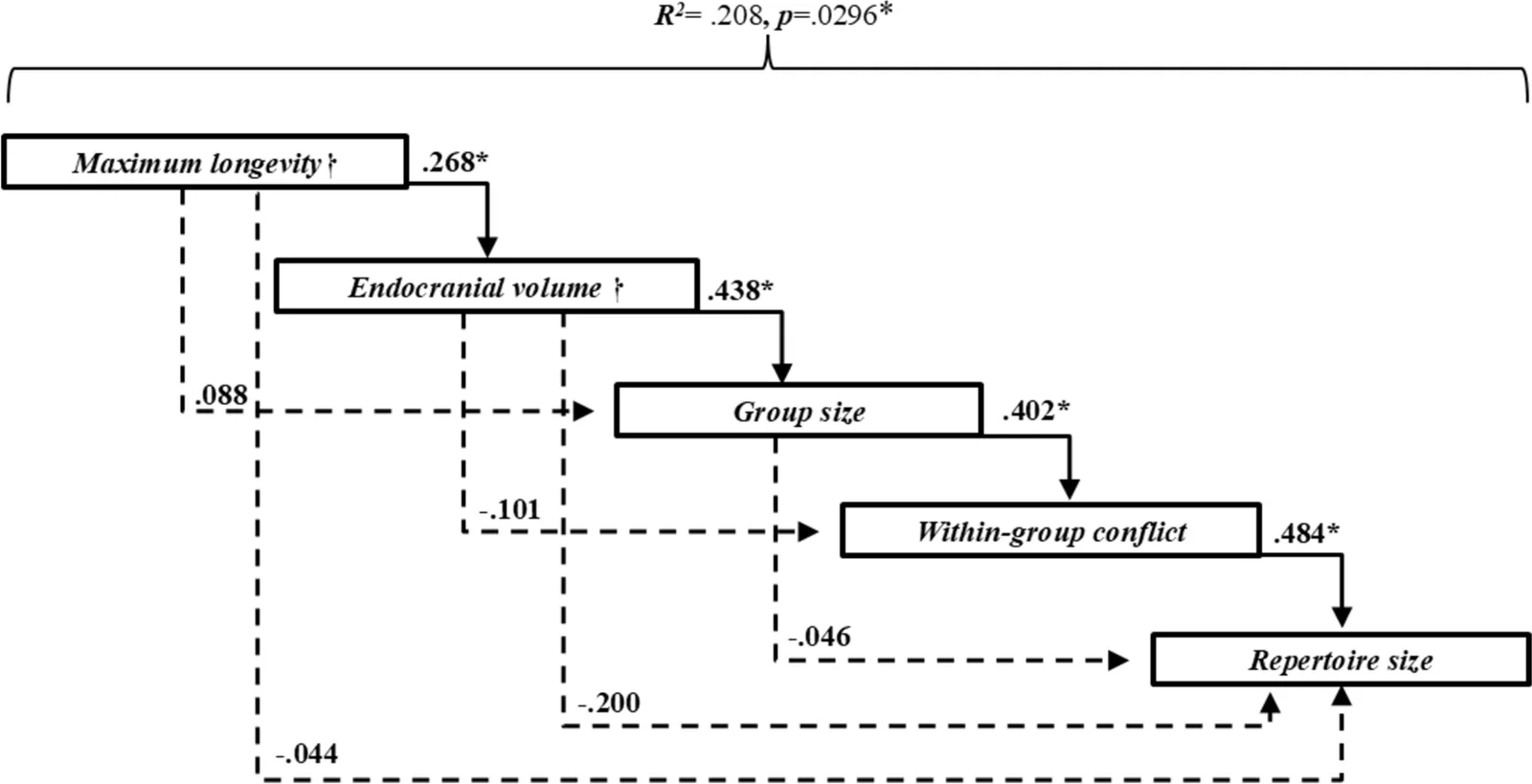
Simplified diagram of a Phylogenetic Sequential Canonical Cascade model featuring statistically significant (*p < .05) standardized direct and indirect influences of life history (*maximum longevity*, *endocranial volume*) and social variables (*group size*, *within-group conflict*) on primate vocal repertoire size (*vocal repertoire size*). The dagger symbol () indicates the variable was also residualized for log (ln) body mass. All variables were residualized for research effort

Based on a PSCCM we tested the following predictions: (1) *maximum longevity* will positively predict *endocranial volume*; (2) *endocranial volume* will positively predict *group size*; (3) *group size* will positively predict *within-group conflict*; and (4) *within-group conflict* will positively predict *vocal repertoire size*. The *maximum longevity* and *endocranial volume* measures in this model also control for *log body mass*, with all variables residualized for research effort. Table 4 and Fig. 2 report and illustrate the results of our PSCCM, which we briefly summarize below. Replicating all analyses with the original McComb & Semple (2005) vocal repertoire counts yields fully consistent and, at the final step, stronger results: within-group conflict predicts vocal repertoire size with β = 0.370, F = 12.14, p = 0.0013, R^2^ = 28.9%, compared to β = 0.487, p = 0.0070, R^2^ = 21.9% in the main analysis (see Appendix). The core finding is therefore robust to the specific repertoire update choices made for individual species and is not weakened by any of the data revisions provided with our updated set of vocal repertoire counts*.*

**Table 4:** A phylogenetic sequential canonical cascade established as a series of phylogenetic generalized linear models, evaluating the influence of maximum longevity, endocranial volume, group size, and within-group conflict factor on the vocal repertoire size of non-human primates

Criterion: endocranial volume				
Phylogenetic signal	kappa [Fix]	lambda [ ML]	lambda l.b (p-value)	lambda u.b (p-value)
	1.000	0.939	0.0004	0.4656

Predictors	Estimate	Std. Error	F-value	Pr(> F)
Maximum Longevity	0.268	0.113	5.57	0.0232

Criterion: Group size				
Phylogenetic signal	kappa [Fix]	lambda [ ML]	lambda l.b (p-value)	lambda u.b (p-value)
	1.000	0.087	0.5404	< 0.0001*

Predictors	Estimate	Std. Error	F-value	Pr(> F)
Endocranial volume	0.438	0.149	10.11	0.0028*
Maximum longevity	0.088	0.142	0.39	0.5338

Omnibus	R^2^	Df	F-value	Pr(> F)
	0.206	(1,40)	10.40	0.0025*

Criterion: within-group conflict				
Phylogenetic signal	kappa [Fix]	lambda [ ML]	lambda l.b (p-value)	lambda u.b (p-value)
	1.000	0.521	0.0208	< 0.0001*

Predictors	Estimate	Std. Error	F-value	Pr(> F)
Group size	0.402	0.134	9.47	0.0038*
Endocranial volume	− 0.101	0.157	0.23	0.6355
Maximum longevity	0.107	0.128	0.729	0.3984

Omnibus	R^2^	Df	F-value	Pr(> F)
	0.197	(2,39)	4.80	0.0137*

Criterion: vocal repertoire size				
Phylogenetic signal	kappa [Fix]	lambda [ ML]	lambda l.b (p-value)	lambda u.b (p-value)
	1.000	0.000	1.000	0.0005*

Predictors	Estimate	Std. Error	F-value	Pr(> F)
Within-group conflict	0.484	0.175	7.57	0.0090*
Group size	− 0.046	0.189	0.72	0.4029
Endocranial volume	− 0.200	0.167	1.61	0.2119
Maximum longevity	− 0.044	0.151	0.09	0.7720

Omnibus	R^2^	Df	F-value	Pr(> F)
	0.208	(3,38)	3.33	0.0296*

Maximum longevity and endocranial volume were residualized for log body size and research effort

All other variables were residualized for research effort

### Ancestral character reconstructions

**Fig. 3:**
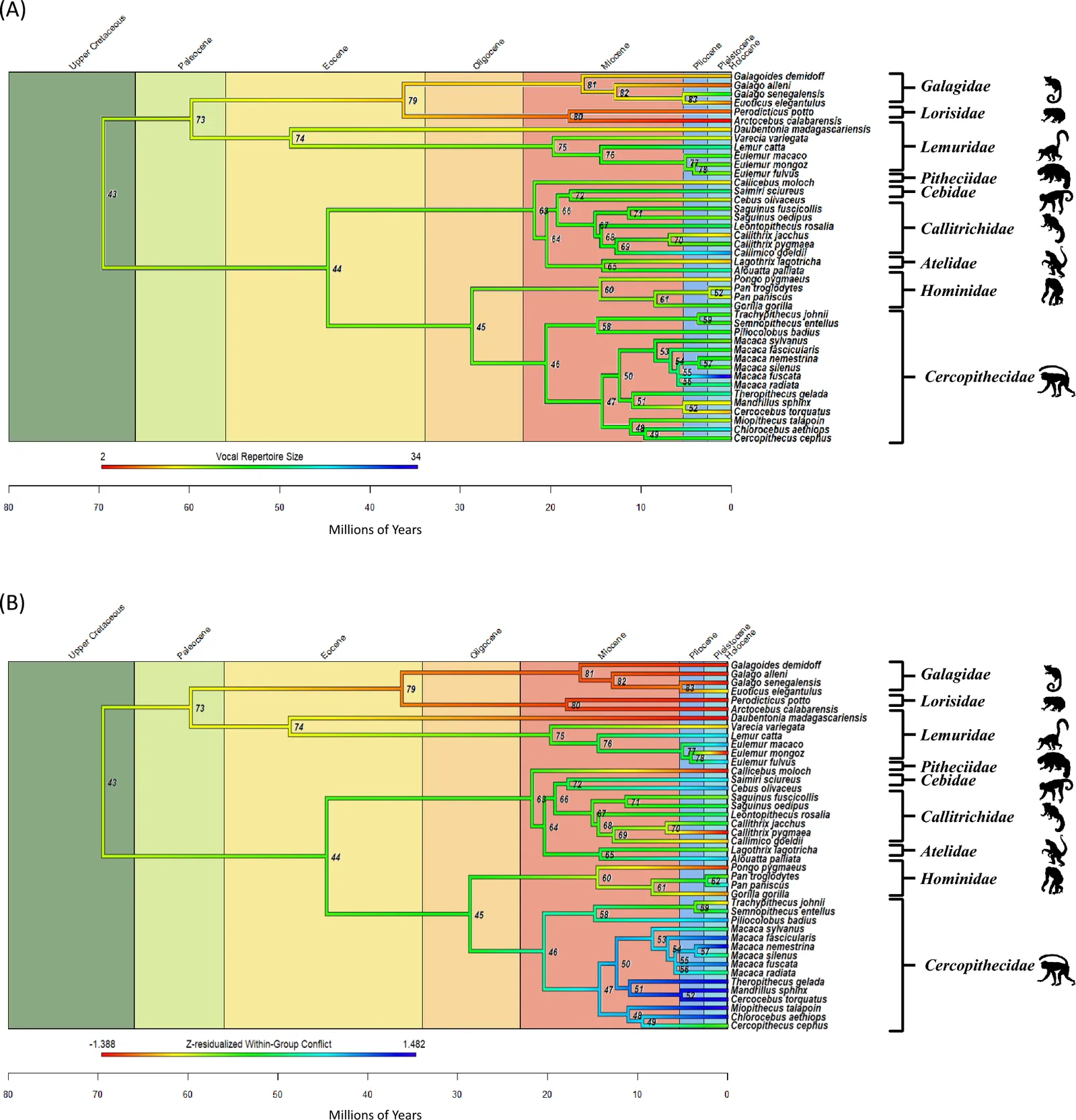
Ancestral character reconstruction (ACR) models. Panel **A** Shows the ACR for vocal repertoire size and panel **B** shows the ACR for within-group conflict (*Z*-scored). For illustration purposes only, reconstructed ancestral within-group conflict and vocal repertoire size are displayed without controls for research effort, to provide measures consistent with the published values represented among extant primates, whereas elsewhere, in our predictive statistical analyses, we have controlled for research effort. Data for ancestral nodes is available online (see https://doi.org/10.5281/zenodo.18291582). The silhouettes of representative primates per taxonomic family were downloaded from http://www.phylopic.org and are in the public domain

With insights from natural history and phylogenetics, we can identify salient macroevolutionary changes in our ancestral character reconstruction (ACR) models among primate clades and across epochs; here we contextualize these trends, noting two major adaptive shifts towards improved diets, slower life histories, and relatively larger brains. Figure 3 shows ACR models of within-group conflict (*Z*-scored) and vocal repertoire size, throughout primate evolution.

Early in primate evolution, the first of the adaptive shifts towards slower life histories and relatively larger brains within the primate order occurred with haplorrhines, whose defining characteristics include a reorganized sensory system dominated by vision as opposed to the olfaction more favoured among strepsirrhines (Heesy and Ross 2001). These cerebral and sensory changes coevolved with haplorrhines’ dietary shift from nocturnal insectivory to a diurnal focus on nutrient-rich, high-energy plant foods like seeds and fruits (Benefit 2000; Fleagle 2013).

In the Miocene, the anthropoid clade underwent a second major shift towards slower life histories and relatively larger brains. During this time, hominids made a dietary shift to dependence on ripe fruits, as evidenced by their suspensory shoulder adaptations that gave their larger bodies access to hanging fruits and freed them from dietary competition with cercopithecoids (Hunt 2016). As an arboreal food, fruits are very nutritious packets high in energy but much more spatially and temporally dispersed than alternatives like foliage. *Among* hominids, *our ACR model identifies general increases in within-group conflict and vocal repertoires* during the Miocene and Pliocene.

*Endocranial volume* was positively and significantly predicted by *maximum longevity* (*β* = 0.268, *F* = 5.57, *p* = 0.0232). This step in the model featured a high joint phylogenetic signal (λ = 0.939) suggesting that the models’ residuals were phylogenetically conserved at the macroevolutionary level.

*Group size* was significantly predicted by *endocranial volume* but not by *maximum longevity* (*β* = 0.438, *F* = 10.11, *p* = 0.0028). This step featured a small phylogenetic signal (λ = 0.087). The omnibus test was statistically significant, with the model explaining 20.6% of the variance.

*Within-group conflict* factor was positively and significantly predicted by *group size* (*β* = 0.402, *F* = 9.47, *p* = 0.0038). This criterion variable was not predicted by *endocranial volume* or *maximum longevity*. The model’s joint phylogenetic signal reached moderate levels (λ = 0.521). The omnibus test was statistically significant and explained 19.7% of the variance.

*Vocal repertoire size* was positively and significantly predicted by *within-group conflict* factor (*β* = 0.484, *F* = 7.57, *p* = 0.0090). Neither *group size* (*p* = 0.4029), *endocranial volume* (*p* = 0.2119), nor *maximum longevity* (*p* = 0.7720), significantly predict vocal repertoire size. The model’s joint phylogenetic signal was low (λ = 0.000). This last step of the model was statistically significant and explained 20.8% of the variance. Within-group conflict also positively and significantly predicted vocal repertoire size when using the original McComb and Semple (2005) dataset (see Appendix).

## Discussion

The phylogenetic sequential canonical cascade model (PSCCM) confirms that within-group conflict directly predicts vocal repertoire size across 42 non-human primate species (Step 4, *R*^*2*^ = 20.8%, *p* = 0.0296). Neither group size (*p* = 0.403), endocranial volume (*p* = 0.212), nor maximum longevity (*p* = 0.772) significantly predict repertoire, while ancestral character reconstructions (ACR) reveal parallel macroevolutionary increases in both traits that are consistent with social conflict and vocal repertoire coevolving under social complexity pressures. Together our results support an embodied capital interpretation, in which slower life histories afford neural investments for managing larger, more conflicted groups, as seen in Miocene-Pliocene shifts among haplorhines and hominids towards higher quality diets that offset brain and vocal costs (Kaplan et al. 2003, 2007; van Schaik and Isler 2012; Hunt 2016). Our results extend McComb & Semple’s (2005) foundational finding of a group size-repertoire link by: revealing within-group conflict as the proximal mediator, (2) incorporating 20 years of updated repertoire data, and (3) integrating life history predictions overlooked in prior studies via the full PSCCM cascade (Peckre et al. 2019; Kavanagh et al. 2021).

Among primates, larger vocal repertoires plausibly help regulate competition and negotiate relationships in groups that experience frequent within-group conflict over patchy, high-value resources. Comparative work also shows that species can invest differently in vocal morphology and usage; for example, some taxa have evolved highly specialized low-frequency vocal anatomy for long-distance signalling (e.g., howler monkeys), whereas others exhibit more flexible combinations and graded uses of calls that support nuanced social communication (e.g., chimpanzees) (Delgado 2006; Leroux et al. 2023).

These contrasts highlight that our repertoire-size measure captures the number of species-typical, acoustically discrete call types, but not how those calls are recombined, modified, or contextually deployed by individuals within or between groups*.* Because features such as call sequencing, recombination, and graded variation can substantially enrich “vocal complexity,” repertoire count should be understood as an imperfect proxy for communicative complexity rather than a comprehensive measure of it (Ouattara et al. 2009; Gustison et al. 2012; Girard-Buttoz et al. 2022, 2025). Non-human primate vocal systems thus represent embodied capital investments that scaffold social coordination, with our results illuminating macroevolutionary preconditions—slower life histories supporting conflict management demands that scale with group size—which likely set the stage for hominin vocal elaboration preceding Pleistocene language emergence. While human language uniquely enables the cultural transmission of fitness-enhancing information through versatile vocalization, gestures, and oral traditions (Kirby et al. 2008; Scalise Sugiyama 2017; Schniter et al. 2018, 2022), the shared primate pathway from social conflict demands to increased vocal repertoire size offers a framework for understanding its deep evolutionary roots.

Our analysis adheres to McComb & Semple (2005) criteria for adult discrete calls, excluding context flexibility, recombination, or non-vocal modalities, which limits insights into full communicative systems (Price et al. 2015; Hammerschmidt and Fischer 2019; Deshpande et al. 2023). Species-level data obscure individual variation, and research effort residuals may not eliminate all biases. Future work should parse call subclasses (e.g., aggression/food: Marler and Tenaza 1977), fission–fusion dynamics (Lehmann and Boesch 2004), and gut morphology trade-offs (Appendix).

## Conclusion

Phylogenetic analyses reveal that within-group conflict directly predicts vocal repertoire size in non-human primates, with life history traits enabling the capacity for embodied capital investment to meet social demands. Ancestral trait estimates suggest many anthropoid species exhibited elevated repertoires during Miocene-Pliocene adaptive shifts, underscoring vocal systems’ role in conflict mediation. This framework elucidates primate vocal evolution and highlights pathways for macroevolutionary research.

## Supplementary Information

Below is the link to the electronic supplementary material.

Supplementary file1 (DOCX 111 KB)

## Data Availability

The data that support the findings of this study are openly available at https://doi.org/10.5281/zenodo.18291582.

## Footnotes

1 Primates invest differentially in embodied communication systems across modalities. Visual displays (coloured pelage, gestures, facial expressions) communicate identity and mate quality—hair colour signals parasite load and hormonal status (West and Packer 2002; Clough et al. 2009; Hofreiter and Schöneberg 2010) and species identity (Santana et al. 2013; Allen et al. 2014). Olfactory systems serve as alternative social information channels (Barton 2006); strepsirrhines possess relatively larger olfactory anatomy and brain regions plus nearly twice the olfactory receptor gene repertoires of haplorhines (Stephan et al. 1981; Martin 1990; Bhatnagar and Meisami 1998; Heesy and Ross 2001; Niimura et al. 2018). Notably, hominin brain expansion correlates with reduced olfactory abilities, muscularity, and gastrointestinal mass (Kaplan et al. 2007).

2 Consistent evidence from birds (Freeberg 2006; Freeberg et al. 2012), lizards (Ord and Martins 2006), sciurid rodents (Blumstein and Armitage 1997; Pollard and Blumstein 2012), bats (Wilkinson 2003), non-human primates (McComb and Semple 2005; Gustison et al. 2012), and humans (Nettle 2012) supports “social complexity” explanations of vocal complexity.

3 Larger groups targeting high-quality, patchy resources (e.g., ripe fruits) exhibit fission–fusion dynamics—repeatedly splitting for dispersed foraging and reforming—which heighten both the frequency and intensity of social conflict over dominance, affiliative access, and mating opportunities (Aureli et al. 2008; Sueur et al. 2011). These dynamics demand cognitive tracking of shifting alliances; in chimpanzees, for example, larger vocal repertoires appear to function in regulating conflict associated with such complex social interactions (Goodall 1986; Mitani and Nishida 1993). However, our dataset does not quantify fission–fusion intensity per se; instead, we operationalize the social complexity arising from these dynamics through group size and within-group conflict (see Lehmann and Boesch 2004; van Schaik 2016).

4 All known languages utilize only a small subset of the many sounds that human speech organs can produce (Moran et al. 2012), though phonemic complexity across languages varies greatly, with humans capable of much larger vocal repertoires (Dryer and Haspelmath 2013; Crystal 2018). A total of 728 phonemes have been identified across 2082 languages worldwide (Creanza et al. 2015).
