# Supplementary material for: Evolution of primate vocal repertoires: vocal systems as embodied capital for mediating within-group conflict
Source: Primates. 2026 Jun 18;67(6):615–28. doi: 10.1007/s10329-026-01271-2 (PMC13731511; doi:10.1007/s10329-026-01271-2)
Supplement: Supplementary file 1 [file 10329_2026_1271_MOESM1_ESM.docx]

**Appendix**

This appendix presents two robustness analyses supporting the main findings on primate vocal repertoire evolution. The first replicates the Phylogenetic Sequential Canonical Cascade Model (PSCCM) using the original McComb and Semple (2005) vocal repertoire dataset. The second examines gut morphology data from Duque-Correa et al. (2021) to test embodied capital trade-offs. Detailed results, data, and Zenodo link follow in the respective sections below.

**PSCCM Replication Analysis with McComb & Semple (2005) Data**

This section presents a brief comparison between the vocal repertoire size values and the estimates obtained in the present study (Table 5). The PSCCM results are based on the original M&S database. For the models based on the updated values, please refer to the main text.

**Table 5.**  Comparison between the 2005 McComb and Semple (“M&S”) vocal repertoire values and the “Updated” vocal repertoire counts gathered from the present study.

| **Species** | **M&S (2005)** | **Updated** | **Change** | **% Change** | **Sample shift** | **Update source** |
| --- | --- | --- | --- | --- | --- | --- |
| *Pan paniscus* | 38 | 11 | −27 | −71.1% | captive → wild | Wegdell et al. 2025 |
| *Pan troglodytes* | 29 | 12 | −17 | −58.6% | wild → wild | Girard-Buttoz et al. 2025; Crockford 2019 |
| *Saguinus oedipus* | 33 | 14 | −19 | −57.6% | captive → wild | Paulo-Morelo & Sánchez-Palomino 2021 |
| *Cercocebus torquatus* | 18 | 8 | −10 | −55.6% | wild → captive | Bouchet et al. 2010 |
| *Callithrix jacchus* | 13 | 8 | −5 | −38.5% | captive → captive | Bosshard et al. 2024 |
| *Eulemur fulvus* | 20 | 14 | −6 | −30.0% | wild → wild | Fichtel & Kappeler 2022; Gamba et al. 2015 |
| *Varecia variegata* | 13 | 11 | −2 | −15.4% | mixed → wild | Batist et al. 2023 |
| *Callicebus moloch* | 11 | 10 | −1 | −9.1% | wild → captive | Muir et al. 2025 |
| *Gorilla gorilla* | 16 | 17 | +1 | +6.2% | — | Salmi et al. 2013 |
| *Leontopithecus rosalia* | 16 | 20 | +4 | +25.0% | captive → wild | Lopes 2020 |
| *Eulemur macaco* | 13 | 16 | +3 | +23.1% | wild → wild | Pozzi et al. 2010; Fichtel & Kappeler 2022 |
| *Eulemur mongoz* | 9 | 15 | +6 | +66.7% | wild → mixed | Nadhurou et al. 2016 |
| *Lagothrix lagothricha* | 6 | 8 | +2 | +33.3% | wild → wild | León et al. 2014 |

Given variability in primate vocal repertoire estimates (e.g., McComb & Semple, 2005), this section re-evaluates the PSCCM using the original McComb and Semple (2005) dataset (see Schniter & Peñaherrera-Aguirre, 2026) to test the robustness of our main findings. The maximum longevity and endocranial volume measures in this model also control for log body mass, with all variables residualized for research effort. Figure 4 and Table 6 report and illustrate these replication results, which we briefly summarize below.

**Figure 4**. Abridged Phylogenetic Sequential Canonical Cascade Model examining the influence of maximum longevity, endocranial volume, group size, and within-group conflict factor on the original vocal repertoire size data published by McComb & Semple (2005).

Endocranial volume was positively and significantly predicted by maximum longevity (*β*= 0.268, *F* = 5.57, *p* = 0.0232). This step in the model featured a high joint phylogenetic signal (λ = 0.939).

Group size was significantly predicted by endocranial volume (*β* = 0.435, *F* = 9.73, *p* = 0.0034) but not by maximum longevity (*p* = 0.6211). The omnibus test was statistically significant (*F* = 9.91, *p* = 0.0031) and explained 19.9% of the variance. The model featured a low joint phylogenetic signal (λ = 0.064).

Within-group conflict factor was positively and significantly predicted by group size (*β*= 0.415, *F* = 10.40, *p* = 0.0026). This criterion variable was not predicted by endocranial volume (*p* = 0.6294) or maximum longevity (*p* = 0.3648). The omnibus test was statistically significant (*F* = 5.318, *p* = 0.0091) and explained 21.4% of the variance. The model featured a moderate joint phylogenetic signal (λ = 0.514).

Vocal repertoire size was positively and significantly predicted by within-group conflict factor (*β*= 0.370, *F* = 12.14, *p* = 0.0013). Neither group size (*p* = 0.2232), endocranial volume (*p* = 0.1988), nor maximum longevity (*p* = 0.7831) significantly predicted vocal repertoire size. The omnibus test was statistically significant (*F* = 5.152, *p* = 0.0044) and explained 28.9% of the variance. The model featured a low joint phylogenetic signal (λ = 0.000).

**Table 6.** A phylogenetic sequential canonical cascade established as a series of phylogenetic generalized linear models, evaluating the influence of maximum longevity, endocranial volume, group size, and within-group conflict factor on the vocal repertoire size of non-human primates, based on the original data published by McComb and Semple (2005). Maximum longevity and endocranial volume were residualized for log body size and research effort. All other variables were residualized for research effort.

| ***Criterion: Endocranial volume*** | |  |  |  |
| --- | --- | --- | --- | --- |
| ***Phylogenetic signal*** | ***kappa [Fix]*** | ***lambda [ ML]*** | ***lambda l.b (p-value)*** | ***lambda u.b (p-value)*** |
|  | 1.000 | 0.939 | 0.0004 | 0.4656 |
| ***Predictors*** | ***Estimate*** | ***Std. Error*** | ***F-value*** | ***Pr(>F)*** |
| *Maximum Longevity* | 0.268 | 0.113 | 5.57 | 0.0232 |
| ***Criterion:*** |  |  |  |  |
| ***Group size*** | |  |  |  |
| ***Phylogenetic signal*** | ***kappa [Fix]*** | ***lambda [ ML]*** | ***lambda l.b (p-value)*** | ***lambda u.b (p-value)*** |
|  | 1.000 | 0.064 | 0.6549 | <0.0001* |
| ***Predictors*** | ***Estimate*** | ***Std. Error*** | ***F-value*** | ***Pr(>F)*** |
| *Endocranial volume* | 0.435 | 0.149 | 9.73 | 0.0034* |
| *Maximum longevity* | 0.071 | 0.144 | 0.25 | 0.6211 |
| ***Omnibus*** | ***R^2^*** | ***Df*** | ***F-value*** | ***Pr(>F)*** |
|  | 0.199 | (1,40) | 9.91 | 0.0031* |
| ***Criterion:*** |  |  |  |  |
| ***Within-group conflict*** |  |  |  |  |
| ***Phylogenetic signal*** | ***kappa [Fix]*** | ***lambda [ ML]*** | ***lambda l.b (p-value)*** | ***lambda u.b (p-value)*** |
|  | 1.000 | 0.514 | 0.0210 | <0.0001* |
| ***Predictors*** | ***Estimate*** | ***Std. Error*** | ***F-value*** | ***Pr(>F)*** |
| *Group size* | 0.415 | 0.134 | 10.40 | 0.0026* |
| *Endocranial volume* | -0.105 | 0.155 | 0.24 | 0.6294 |
| *Maximum longevity* | 0.114 | 0.126 | 0.84 | 0.3648 |
| ***Omnibus*** | ***R^2^*** | ***Df*** | ***F-value*** | ***Pr(>F)*** |
|  | 0.214 | (2,39) | 5.318 | 0.0091* |
| ***Criterion:*** |  |  |  |  |
| ***Vocal repertoire size*** |  |  |  |  |
| ***Phylogenetic signal*** | ***kappa [Fix]*** | ***lambda [ ML]*** | ***lambda l.b (p-value)*** | ***lambda u.b (p-value)*** |
|  | 1.000 | 0.000 | 1.000 | 0.0001* |
| ***Predictors*** | ***Estimate*** | ***Std. Error*** | ***F-value*** | ***Pr(>F)*** |
| *Within-group conflict* | 0.370 | 0.167 | 12.14 | 0.0013* |
| *Group size* | 0.297 | 0.180 | 1.53 | 0.2232 |
| *Endocranial volume* | -0.194 | 0.157 | 1.71 | 0.1988 |
| *Maximum longevity* | -0.040 | 0.143 | 0.07 | 0.7831 |
| ***Omnibus*** | ***R^2^*** | ***Df*** | ***F-value*** | ***Pr(>F)*** |
|  | 0.289 | (3,38) | 5.152 | 0.0044* |

**Analysis of Gut Morphology and Brain Trade-offs**

To evaluate alternative datasets for costly embodied capital traits, we analysed gut morphology (*caecum length*, *colon length*, *large intestine length*, *small intestine length*) from Duque-Correa et al. (2021) to assess how dietary quality shapes trade-offs, where complex guts for low-quality diets constrain brain and vocalization system investments, while simpler guts for high-quality diets enable larger brains and expanded vocal repertoires. These variables were residualized for the allometric of log body size and research effort and are available at Zenodo.org, [DOI 10.5281/zenodo.18291582](https://doi.org/10.5281/zenodo.18291582). The corresponding residuals were examined via several Phylogenetic Generalized Least Squares models. These analyses revealed a negative and significant influence of the standardized and residualized large intestine size (*β* = -0.447, *p* = 0.0179) and the caecum size (*β* = -0.714, *p* = 0.0009) on the endocranial volume values. In contrast, there was no significant influence of the standardized and residualized small intestine size (*β* = 0.150, *p* = 0.4585), and colon size (*β* = -0.332, *p* = 0.0621) on the endocranial volume values. These results indicate that, even with a reduced set of 21 primate species, as examined in the present study, several negative associations emerge between various components of somatic capital, as exemplified by the gut-brain trade-off. These models demonstrate that gut-brain trade-offs, shaped by dietary quality, converge with the main model’s findings, supporting the embodied capital hypothesis linking costly traits to social complexity and vocal repertoire size in primates.

**References**

Di Fiore, A., Link, A., & Campbell, C. J. (2007). The atelines: Variation in ecology, behavior, and social organization. In C. J. Campbell, A. Fuentes, K. C. MacKinnon, M. Panger, & S. K. Bearder (Eds.), *Primates in perspective* (pp. 155–185). Oxford University Press.

Digby, Ferrari, and Saltzman (2007), Jack (2007), Norconk (2007), Fernández-Duque (2007), Di Fiore, Link, and Campbell (2007),

Digby, L. J., Ferrari, S. F., & Saltzman, W. (2007). Callitrichines: The role of competition in cooperatively breeding species. In C. J. Campbell, A. Fuentes, K. C. MacKinnon, M. Panger, & S. K. Bearder (Eds.), *Primates in perspective* (pp. 85–105). Oxford University Press.

Duque-Correa, M. J., Codron, D., Meloro, C., McGrosky, A., Schiffmann, C., Edwards, M. S., & Clauss, M. (2021). Mammalian intestinal allometry, phylogeny, trophic level and climate. Proceedings of the Royal Society B: Biological Sciences, 288(1944), Article 20202888. https://doi.org/10.1098/rspb.2020.2888

Fashing, P. J. (2007). African colobine monkeys: Patterns of between-group interaction. In C. J. Campbell, A. Fuentes, K. C. MacKinnon, M. Panger, & S. K. Bearder (Eds.), *Primates in perspective* (pp. 201–224). Oxford University Press.

Fernández-Duque, E. (2007). Aotinae: Social monogamy in the only nocturnal haplorhines. In C. J. Campbell, A. Fuentes, K. C. MacKinnon, M. Panger, & S. K. Bearder (Eds.), *Primates in perspective* (pp. 139–154). Oxford University Press.

Gould, L., & Sauther, M. (2007). Lemuriformes. In C. J. Campbell, A. Fuentes, K. C. MacKinnon, M. Panger, & S. K. Bearder (Eds.), *Primates in perspective* (pp. 46–63). Oxford University Press.

Jack, K. M. (2007). The cebines: Toward an explanation of variable social structure. In C. J. Campbell, A. Fuentes, K. C. MacKinnon, M. Panger, & S. K. Bearder (Eds.), *Primates in perspective* (pp. 107–123). Oxford University Press.

Jaffe, K. E., & Isbell, L. A. (2007). The guenons: Polyspecific associations in socioecological perspective. In C. J. Campbell, A. Fuentes, K. C. MacKinnon, M. Panger, & S. K. Bearder (Eds.), *Primates in perspective* (pp. 277–300). Oxford University Press.

Kirkpatrick, R. C. (2007). The Asian colobines: Diversity among leaf-eating monkeys. In C. J. Campbell, A. Fuentes, K. C. MacKinnon, M. Panger, & S. K. Bearder (Eds.), *Primates in perspective* (pp. 186–200). Oxford University Press.

McComb K, Semple S (2005) Coevolution of vocal communication and sociality in primates. Biology Letters 1:381–385. <https://doi.org/10.1098/rsbl.2005.0366>

Norconk, M. A. (2007). Sakis, uakaris, and titi monkeys: Behavioral diversity in a radiation of primate seed predators. In C. J. Campbell, A. Fuentes, K. C. MacKinnon, M. Panger, & S. K. Bearder (Eds.), *Primates in perspective* (pp. 123–138). Oxford University Press.

Schniter, E., & Peñaherrera-Aguirre, M. (2026). Data Associated with Manuscript Titled "Evolution of Primate Vocal Repertoires: Vocal Systems as Embodied Capital for Mediating Within-group Conflict" [Data set]. Zenodo. [DOI 10.5281/zenodo.18291582](https://doi.org/10.5281/zenodo.18291582).

Swedell, L. (2007). Baboons, mandrills, and mangabeys: Afro-papionin socioecology in a phylogenetic perspective. In C. J. Campbell, A. Fuentes, K. C. MacKinnon, M. Panger, & S. K. Bearder (Eds.), *Primates in perspective* (pp. 240–256). Oxford University Press.
